# Therapeutic Targeting of Histone Modifications in Adult and Pediatric High-Grade Glioma

**DOI:** 10.3389/fonc.2017.00045

**Published:** 2017-03-28

**Authors:** Maria J. Williams, Will G. B. Singleton, Stephen P. Lowis, Karim Malik, Kathreena M. Kurian

**Affiliations:** ^1^Brain Tumour Research Group, Institute of Clinical Neurosciences, University of Bristol, Bristol, UK; ^2^Functional Neurosurgery Research Group, Institute of Clinical Neurosciences, University of Bristol, Bristol, UK; ^3^Department of Paediatric and Adolescent Oncology, Bristol Royal Hospital for Children, Bristol, UK; ^4^Cancer Epigenetics Laboratory, Cellular and Molecular Medicine, University of Bristol, Bristol, UK

**Keywords:** glioblastoma multiforme, diffuse intrinsic brainstem glioma, histone methylation, histone acetylation, histone deacetylase inhibitors, epigenetics, high-grade glioma

## Abstract

Recent exciting work partly through The Cancer Genome Atlas has implicated epigenetic mechanisms including histone modifications in the development of both pediatric and adult high-grade glioma (HGG). Histone lysine methylation has emerged as an important player in regulating gene expression and chromatin function. Lysine (K) 27 (K27) is a critical residue in all seven histone 3 variants and the subject of posttranslational histone modifications, as it can be both methylated and acetylated. In pediatric HGG, two critical single-point mutations occur in the H3F3A gene encoding the regulatory histone variant H3.3. These mutations occur at lysine (K) 27 (K27M) and glycine (G) 34 (G34R/V), both of which are involved with key regulatory posttranscriptional modifications. Therefore, these mutations effect gene expression, cell differentiation, and telomere maintenance. In recent years, alterations in histone acetylation have provided novel opportunities to explore new pharmacological targeting, with histone deacetylase (HDAC) overexpression reported in high-grade, late-stage proliferative tumors. HDAC inhibitors have shown promising therapeutic potential in many malignancies. This review focuses on the epigenetic mechanisms propagating pediatric and adult HGGs, as well as summarizing the current advances in clinical trials using HDAC inhibitors.

## Introduction

Recent exciting work partly through The Cancer Genome Atlas (TCGA), has implicated epigenetic mechanisms including histone modifications in the development of both pediatric and adult high-grade glioma (HGG). Importantly, epigenetic modifications have a potential for novel therapeutic drug targeting as epigenetic changes are catalyzed by highly specific enzyme complexes. For the purpose of this review, the term HGG is used to describe the astrocytic gliomas: anaplastic astrocytoma (WHO Grade III) and glioblastoma (WHO Grade IV) (1). The term diffuse intrinsic pontine glioma (DIPG) refers to a specific astrocytic glioma, which arises in the ventral pons in children who carries a uniformly fatal prognosis, with a median survival of 9 months (1). The annual incidence of adult glioblastoma is 7.2 per 100,000, making it the most common adult primary intrinsic brain tumor (2). By contrast, in children, HGGs are reported to have an annual incidence of 0.8 per 100,000, representing 8–12% of pediatric central nervous system tumors and making them rare compared to other tumor types (2–4).

Unlike genetic abnormalities, epigenetic abnormalities encompass modifications that do not result from a change in the primary DNA sequence (5). These modifications include DNA methylation, histone methylation, and acetylation as well as other modifications that can indirectly regulate gene expression (5). A classic epigenetic modification in adult glioblastoma is DNA hypermethylation of the enzyme O-6-methylguanine-DNA methyltransferase, which suppresses its normal function to remove alkyl groups from DNA (6). This makes such patients more sensitive to alkylating agents such as temozolomide (6).

Histones are positively charged proteins namely H1, H2A, H2B, H3, and H4, which make up the macromolecular three-dimensional complex of chromatin together with negatively charged DNA (7). The nucleosome is the fundamental subunit of chromatin comprising a histone octamer with two copies of each of histones H2A, H2B, H3, and H4 (7). Histone H3 has seven known sequence variants in mammalian cells, which are highly sequence conserved, differing only by a few amino acids. These are histones H3.1, H3.2, H3.3, H3.4 (H3T), H3.5, H3.X, and H3.Y (8). Histone H3.3 has been shown to function in maintaining genome integrity during mammalian development, by supporting chromosomal chromatin structures (9). This histone variant is known to modulate specific chromatin changes and gene expression profiles and is often considered a mark of transcriptional activity (10).

Chromatin remodeling or modification represents a highly dynamic process in which there is continual laying down and removal of modifications by chromatin-remodeling enzymes resulting in three-dimensional changes, which can affect gene expression by regulating access to RNA polymerases and transcription factors (11). In particular, the *N*-terminal tails of histones contain lysine (K) and arginine (R) residues that can undergo posttranslational modifications including acetylation, methylation, ubiquitylation, and sumoylation, as well as serines that can be phosphorylated (11). These complex modifications affect almost all DNA-dependent processes, including gene expression, DNA replication and repair, and centromere and telomere maintenance (11, 12). Therefore, cross-talk between modifications may lead to myriad read-outs, which are beyond the scope of this review. We will therefore focus mainly on the posttranslational modification of histones by methylation and acetylation, as these are of current clinical interest in adults and pediatric HGG, and both processes are novel pharmacological targets with recent early phase clinical trials.

## Histone Methylation in Pediatric Glioma

### *H3F3A* K27M and G34R/V Histone Mutations in Pediatric HGG

**Figure 1:**
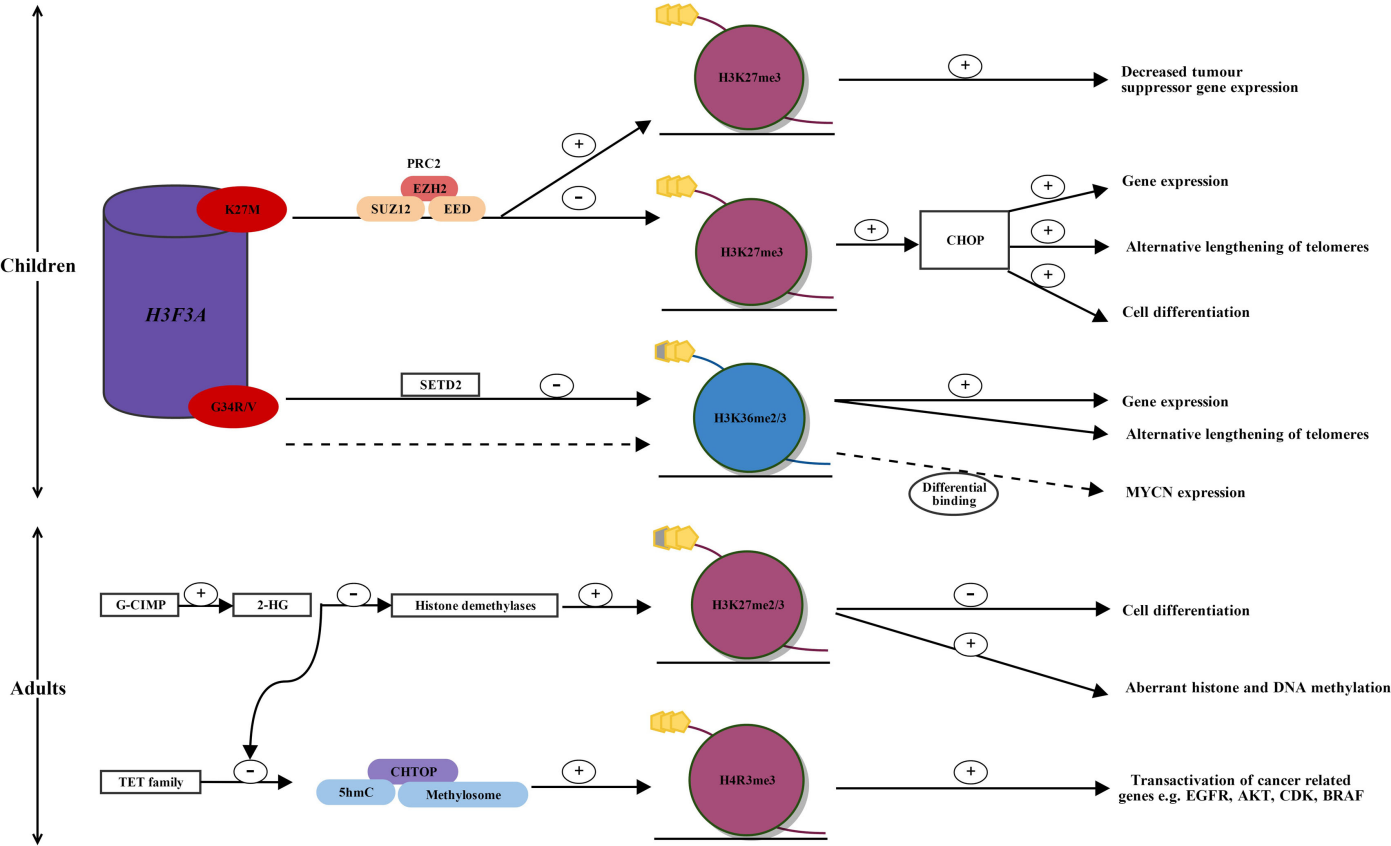
**Alterations in histone methylation in pediatric and adult high-grade glioma**. In children, two single-point mutations in the regulatory histone, *H3F3A*, occur in the histone tail at H3.3 K27M and G34R/V, affecting key regulatory posttranscriptional modifications. H3.3 K27M mutated glioblastoma displays reprogramming of H3K27 methylation. K27M alters the enzymatic activity of EZH2, the catalytic subunit of PRC2, which establishes H3K27 methylation. This leads to a global reduction in H3K27 methylation and the CHOP, priming for increased gene expression, cell differentiation, and alternative lengthening of telomeres. Within a globally hypomethylated phenotype, K27M mutated glioma may allow increased H3K27 methylation at specific gene loci. An increased H3K27 methylation silences tumor suppressor gene expression, such as *p16INKA*. In H3.3 G34R/V mutated glioma, mutations in SETD2 lead to decreased H3K36 methylation, which results in increased gene expression and alternative lengthening of telomeres. MYCN is upregulated through differential genomic binding of methylated H3K36 in G34R/V mutated glioblastoma. In adults, mutated IDH1 and induction of the G-CIMP phenotype lead to the overproduction of 2-HG. 2-HG inhibits histone demethylases leading to increased H3K27 methylation, which leads to a block in cell differentiation, and aberrant DNA and histone methylation. Over production of 2-HG also inhibits ten-eleven translocation (TET) activation, leading to a decrease in the 5hmC/CHTOP/methylosome complex, which is normally present in wild-type IDH1 glioma. This results in decreased transactivation of cancer-related genes such as EGFR, AKT, CDK, and BRAF and may provide an explanation for increased survival in patients with IDH1 mutated glioblastoma. AKT, protein kinase B; BRAF, B-Raf proto-oncogene, serine/threonine kinase; CDK, cyclin-dependant kinase; CHOP, CpG hypomethylator phenotype; CHTOP, chromatin target of PRMT1; EGFR, epidermal growth factor receptor; EZH2, enhancer of zeste homologue 2; G-CIMP, glioma-CpG-island methylator phenotype; G34R/V, glycine34 arginine/valine; *H3F3A*, H3 histone family 3A; H3K27me2/3, histone 3 lysine 27 dimethylation/trimethylation; H4R3, histone 4 arginine 3; 2-HG, 2-hydroxyglutarate; 5hmC, 5-hyroxymethylcytosine; K27M, lysine 34 methionine; PRC2, polycomb repressive complex 2; SETD2, SET domain-containing 2; TET family, ten-eleven translocation family; +, increased; −, decreased; dotted line, alternative pathway in G34R/V mutated glioma.

Schwartzentruber et al. were the first to report recurrent mutations of a regulatory histone, *H3F3A*, in humans by exome sequencing of pediatric glioblastomas (see Figure 1) (13). The histone *H3F3A* encodes the histone variant H3.3, which is predominantly incorporated into transcription sites and telomeric regions, and is associated with active and open chromatin (14). Mutations in *H3F3A* involve two critical single-point mutations in the histone tail at lysine (K) 27 (K27M) and glycine (G) 34 (G34R/V), both of which are involved with key regulatory posttranscriptional modifications (13). As well as being reported in pediatric HGGs, H3.3 mutation are also reported in other childhood cancers such as chondroblastomas and giant cell tumors of the bone (15).

Histone lysine methylation has emerged as an important player in regulating gene expression and chromatin function. K27 is a critical residue in all seven histone 3 variants, and it can be posttranslationally methylated or acetylated (16). Acetylation may induce active transcription, while monomethylation, dimethylation or trimethylation of K27, catalyzed by the histone methyltransferase enhancer of zeste homologue 2 (EZH2), are repressive marks associated with gene silencing (5). The K27M mutation in certain cases results in decreased dimethylation and trimethylation of H3K27 and is associated with transcriptional activation; however, there are alternative mechanisms by which the K27M increases trimethylation, thus silencing tumor suppressor gene expression (17). This occurs through bivalent domains, which occur on histone proteins and allow epigenetic regulators such as methylating enzymes to silence or activate gene expression (17). Similarly, the G34R/V mutation results in redistribution of H3K36 methylation and altered gene transcription, including upregulation of the *MYCN* (V-Myc avian myelocytomatosis viral oncogene neuroblastoma-Derived Homolog) oncogene (13, 18, 19).

Importantly, *H3F3A* mutations have been reported to have 100% specificity for pediatric HGGs, with no evidence of the mutations in pediatric low-grade gliomas, embryonal tumors, or ependymomas (20). Furthermore, several groups have reported that these histone H3.3 mutations are not identified in adult glioblastoma (20–22). Moreover, K27M and G34R/V mutations are associated with differing age and tumor location in childhood HGGs (13, 16, 23). K27M histone H3.3 mutations occur more commonly in younger children (median age 10.5 years, range 5–23 years) and are present in 70–80% of midline brainstem and thalamic glioblastoma (13, 16, 23). G34R/V histone H3.3 mutations have been shown to occur more frequently in older children (median age 18 years, range 9–42 years) and are observed almost exclusively in hemispheric gliomas (16, 23).

The K27M histone H3.3 mutation is associated with a shorter clinical survival [0.73 years (±0.48)] (*p* = 0.0008) compared with patients lacking the mutation [4.59 years (±5.55)]. However, this poor survival may in part reflect the brainstem and midline locations of these pediatric gliomas (16).

### K27M Mutated Pediatric HGG, Polycomb Repressive Complex 2 (PRC2), and the Global Hypomethylator Phenotype

The PRC2 is one of the two complexes of polycomb group proteins; the other component of this group of proteins is the polycomb repressive complex 1 (PRC1). Both PRC2 and PRC1 are often needed to maintain gene repression (24). PRC2 is required for the initial targeting of the genomic region [PRC response elements] to be silenced, while PRC1 is thought to work downstream of PRC2 and stabilizes the cellular memory of the silenced region after cellular differentiation (24).

The PRC2 complex has histone methyltransferase activity and silences gene expression by dimethylating or trimethylating H3K27 through its enzymatic subunits, enhancer of zeste homolog 1 and 2 (EZH1 and EZH2) (24, 25). Lewis et al. reported that PRC2 is inhibited by aberrant binding of mutant K27M to EZH2 (26). Moreover, K27M alters the enzymatic activity of EZH2, the catalytic subunit of PRC2, which establishes H3K27 methylation, thereby leading to a global reduction of H3K27 methylation and the loss of gene repression (see Figure 1) (26, 27).

Bender et al. used chromatin immunoprecipitation, next-generation sequencing, and whole-genome bisulfite sequencing on primary HGGs, to show reduced methylation of H3K27 primes generally for global DNA hypomethylation (28). This leads to the CpG hypomethylator phenotype (CHOP), resulting in the activation of gene expression and cell differentiation (see Figure 1) (28).

Subsequently, although mutant K27M results in a global reduction of H3K27 methylation in HGGs, Chan et al. have reported that at specific gene loci there is a dramatic increase in H3K27 methylation, as well as an increase in the catalytic subunit of the PRC2 histone methyltransferase EZH2 (17, 29). Although DNA hypomethylation promotes gene expression, it would be disadvantageous to tumor cells if this included the expression of genes protecting against tumorigenesis, such as tumor suppressor genes. Therefore, Chan et al. have proposed that by inducing a globally hypomethylated phenotype (CHOP), this allows for increased binding of PRC2 and establishment of H3K27 methylation at specific gene loci (29). It is likely that tumor formation in H3.3 K27M mutated HGGs is driven by chromatin modifications occurring due to the loss and gain of H3K27 methylation at different gene loci (see Figure 1) (29). This correlates, for example, with a gain of H3K27 methylation at *p16INKA4A* and decreased expression of this tumor suppressor gene, consistent with promotion of tumorigenesis (see Figure 1) (29).

### Upregulation of MYCN in G34R/V Mutated Pediatric HGG

By a similar mechanism to H3K27, histone H3K36 is also subject to alterations in methylation (30). Although still occurring in pediatric gliomas with mutations in the histone tail of H3.3, the mutation is G34R/V as opposed to K27M. Fontebasso et al. conducted whole exome sequencing on 60 pediatric HGGs and compared them to 543 non-cancer control samples (30). They showed that decreased methylation of H3K36 has been shown to occur through loss of function mutations in the H3K36 methyltransferase SET domain-containing 2 (30). The decrease in H3K36 trimethylation was shown to correlate with increased gene expression (30).

In addition, the G34R/V mutation was shown to upregulate *MYCN*, with increased RNA polymerase II binding and transcriptional upregulation at the gene locus, through the differential genomic binding of methylated H3K36 to specific gene loci (see Figure 1) (19, 31). MYCN is a potent oncogene implicated in many cancers, often signaling an aggressive and undifferentiated phenotype. Of recent discovery, forced overexpression of *MYCN* has been shown to cause glioblastoma in the developing mouse forebrain, providing evidence for a tumor-initiating event that may drive pediatric glioblastoma formation during neurological development (19, 32).

These insights provide opportunities for novel ways to target specific genetic and epigenetic aberrations in H3.3 G34R/V mutated pediatric HGGs.

### Putative Telomere Maintenance in G34R/V Mutated Pediatric HGG

G34R/V mutations occurring in hemispheric pediatric HGG, frequently display mutations in *TP53, ATRX* (α-thalassemia/mental retardation syndrome X-linked), and *DAXX* (death domain-associated protein), unlike the K27M mutated HGGs (13, 16, 23, 33). Schwartzentruber et al. reported 100% of patients with H3.3 mutated G34R/V glioblastoma and who had mutations in *ATRX* and *DAXX*, which encode two subunits required for H3.3 incorporation at centromeres and telomeres (13, 34, 35).

Pathak et al. further investigated mutations in the H3.3-ATRX-DAXX chromatin remodeling pathway in pediatric glioblastoma (36). They reported a global loss of histone methylation in 80% of cases, particularly a loss of trimethylation in histones H3K27 and H3K4 (36). The combinatorial methylation loss of these histones was associated with *H3F3A-ATRX* mutations, with 60% of K27M cases and 75% of G34R mutant cases displaying *ATRX* loss (36).

*H3F3A/ATRX-DAXX/TP53* mutations are strongly associated with alternative lengthening of telomeres, a telomerase-independent telomere maintenance mechanism that could allow unlimited cellular proliferation in pediatric glioblastoma (13, 33, 37, 38).

## Histone Methylation in Adult Glioma

### The Hypermethylator Phenotype Glioma-CpG-Island Methylator Phenotype (G-CIMP) Is Distinct from *H3F3A* Mutations, Occurring Rarely in Pediatric Glioma but More Frequently in Young Adults

A high proportion of low-grade gliomas and secondary glioblastomas have been shown to harbor mutations in isocitrate dehydrogenase 1 and 2 (*IDH1/IDH2)* (see Figure 1) (39). IDH1 is an enzyme involved in the Krebs cycle of glucose metabolism (40). Its usual function is to decarboxylate isocitrate to yield α-ketoglutarate (40). The mutation of IDH1 results in loss of normal enzymatic function and leads to the abnormal production of 2-hydroxyglutarate (2-HG) (40). 2-HG has been found to inhibit histone and DNA demethylases, causing widespread changes in histone and DNA methylation and potentially promoting tumorigenesis (40).

Although *IDH1* mutations are relatively uncommon in pediatric glioblastoma, single amino acid substitutions of arginine result in gain of function mutations in *IDH1* [commonly arginine to histidine (R132H)] and occur frequently in young adults with secondary glioblastoma, which has progressed from lower grade neoplasms (41, 42). *IDH1* mutational status has been shown to be a positive prognosticator for survival in patients with glioblastoma. IDH1 indirectly affects H3K27 or H3K36 methylation by the oncometabolite 2-HG (43). 2-HG inhibits histone demethylases and is associated with a distinct G-CIMP, specifically increasing methylation of H3K27 and H3K36, which has been associated with a block in cell differentiation (see Figure 1) (43–45). Furthermore, the *IDH1* mutations promoting methylation are associated with mutations in *TP53* (13). This represents a third subgroup of pediatric and young adult glioblastoma, which is mutually exclusive from *H3F3A* mutations (23). The importance of identifying *IDH1* mutation status is important for prognosis in this subgroup, with the opportunity to explore 2-HG inhibition and the potential to prevent the transformation of a low-grade malignancy to a HGG (43).

### Mutation-Independent Downregulation of H3.3 Favors Self-Renewal in Adult Glioblastoma Cancer Cells

Recent breakthrough by Dirk et al. has provided a possible explanation for the paucity of H3.3 mutations in adult glioblastoma, by mutation-independent abnormalities in histone biology (46). By using patient-derived glioblastoma primary cultures that enrich for cells with tumor-initiating and self-renewal potential, they demonstrated that DNA methylation profiles in *H3F3A* wild-type adult glioblastoma were similar to DNA methylation profiles of H3.3 mutated pediatric glioblastoma (46).

Mixed lineage leukemia 5 (MLL5) is the most divergent member of the MLL family. Its biological role is not fully characterized; however, it is known to lack methyltransferase function (47). To identify if epigenetic modifiers are implicated in the downregulation of H3.3 in adult glioblastoma, Dirk et al. investigated gene expression profiling across glioblastoma self-renewing cultures and found *MLL5* to be expressed robustly (46). Overexpression of *MLL5* in glioblastoma primary cultures led to decreased H3.3, while knockdown of *MLL5* led to increased H3.3 protein, showing a direct role for MLL5 in repressing H3.3 (46). Furthermore, *MLL5* overexpression led to increased expression of two chromatin condensing genes; therefore, MLL5 may exert transcriptional repression of H3.3 (*via H3F3B*) by altering chromatin configuration and accessibility (46).

Finally, histone demethylase inhibitors were tested in glioblastoma primary cultures and were shown to have potent suppressive effects of glioblastoma self-renewal (46). These findings suggest that chromatin remodeling could be exploited as a novel therapeutic target in adult glioblastoma.

### Overproduction of 2-HG Inhibits the Ten-Eleven Translocation (TET) Family and H3K27 Demethylases in Adult HGG

Histone modifications occur less commonly in adults compared to children; however, mechanisms of glioma propagation have been explored with regards to H3K27 and histone 4 arginine 3 (H4R3) methylation. Epigenetic modifications of histone proteins occur through *IDH1* mutation and induction of the G-CIMP phenotype (48). Subsequent overproduction of 2-HG inhibits the TET family of 5-hyroxymethylcytosine (5hmC) hydroxylases leading to a decrease in 5hmC (49). 2-HG also inhibits H3K27 demethylases leading to an increase in H3K27 methylation, with resulting aberrant histone and DNA methylation, as well as a block in cell differentiation (see Figure 1) (43, 45).

Taiki et al. showed that glioblastoma cells with wild-type IDH1 contain increased TET and 5hmC levels, and TET-1 production of 5hmC is required for gliomagenesis, by recruiting the chromatin target of PRMT1–methylosome complex (50). 5hmC has been previously reported to act as an intermediate for DNA demethylation but instead recruits DNA-binding proteins (50, 51). The methylosome is an arginine methyltransferase complex that promotes PRMT1-mediated methylation of H4R3 in genes involved in gliomagenesis, including *EGFR, AKT3, CDK6, CCND2*, and *BRAF* (see Figure 1) (50). This provides a mechanism by which patients with mutated *IDH1* have a better survival compared to those with wild-type *IDH1* (52, 53).

### Histone Acetylation in Pediatric and Adult HGG—A Balancing Act between Histone Acetyltransferases (HATs) and Histone Deacetylases (HDACs)

As well as undergoing methylation, histone tails can be also posttranslationally modified by acetylation (54). The balance of action between HATs and HDACs is a key regulatory mechanism in the transcriptional activation and repression of gene expression, respectively (54). HDACs are overexpressed in many cancers, and targeting with HDAC inhibitors has provided a promising avenue in the development of new therapeutic approaches (55). HDACs facilitate the condensation of chromatin, by removing acetyl groups from the *N*-terminal tails of histone proteins, preventing the access of transcriptional machinery to DNA, and by binding of SWI3, ADA2, N-CoR and TFIIIB (SANT) containing proteins (56). SANT containing proteins bind unacetylated proteins, inhibiting HATs and facilitating HDAC binding (54, 56). There are 18 HDAC enzymes, and these are classified as zinc dependent (class I, IIa, IIb, and IV) or zinc independent and NAD dependent (class III) (55). It is particularly class I and II HDACs, which have become the focus for targeting as anticancer therapies (55). Class I HDACs associate with multiprotein complex repressors and have a role in cell survival and proliferation (55, 57–60). Class IIa HDACs have tissue-specific roles, and class IIb HDACs have been identified in the regulation of cell survival in response to stress (58, 61). There are non-histone protein targets of HDACs, including hormone receptors, transcription factors, and DNA repair enzymes (60, 62). The recent advances in the targeting of HDACs with HDAC inhibitors is therefore a complex process due to the lack of HDAC inhibitor specificity for histone proteins and little understanding of their mechanism of action (60, 62, 63).

### Altered Expression of HDACs in Adult and Pediatric Glioblastoma

The Cancer Genome Atlas used genome-wide sequencing of 284 glioblastoma samples to identify somatic mutations in genes involved with histone modifications and identified somatic mutations in *HDAC2* (64).

Lucio-Etevoric et al. evaluated mRNA expression of class I, II, and IV HDACs in 20 low-grade gliomas (13 grade I and 7 grade II) and 23 HGGs (5 grade III and 18 glioblastomas), with patient ages ranging from 1.3 to 79 years (mean age 24.6 years, SD of 12.8 ± 22.6 years) (65). They reported hypoexpression of HDACs II and IV in glioblastoma compared to low-grade gliomas and normal brain tissue (65). In contrast, HDAC I overexpression has been reported in high-grade, late-stage proliferative tumors, supporting the rationale for the use of HDAC inhibitors in promoting the re-expression of silenced tumor suppressor genes in glioblastoma, as well as a more open chromatin structure facilitating access for DNA damaging agents (66, 67). Therefore, HDACs may have a role in repressing genes associated with gliomagenesis, and HDAC inhibitors may not be effective in all cases of glioblastoma, as they may potentiate the transcriptional activation of proto-oncogenes (65, 68).

## Pharmacological Targeting of Histone Modifiers in HGG

### Pediatric HGG Trials Using HDAC Inhibitors

Tables 1 and 2 summarize completed phase I and phase II trials investigating the use of HDAC inhibitors, in pediatric and adult gliomas. The Children’s Oncology Group has conducted two trials investigating the use of the pan-HDAC inhibitor vorinostat as a therapy for pediatric HGG (69, 70). They investigated vorinostat administered singly or in combination 13-cis retinoic acid (isotretinoin) in children with refractory solid tumors (69). They showed the maximum tolerated dose (MTD) of vorinostat was 230 mg/m^2^/dose as a single agent or 180 mg/m^2^/dose 4× per week with and 13cRA 80 mg/m^2^/dose 2× daily, days 1–14 every 28 days (69). Dose-limiting toxicities for vorinostat as a single agent included neutropenia, thrombocytopenia, and hypokalemia (69). Dose-limiting toxicities for vorinostat and 13cRA included neutropenia, thrombocytopenia, anorexia, and hypertriglyceridemia (69). Prolonged stable disease was observed in five patients including one of seven with DIPG, and a complete response was observed in one patient with neuroblastoma (69).

**Table 1:** **Summary of completed phase I clinical trials investigating histone deacetylase inhibitors for the treatment of adult and pediatric high grade glioma**.

Clinical trial	Phase	Population	Results		Clinical observations	Reference
			Maximum tolerated dose	Dose-limiting toxicities		
Vorinostat or vorinostat and 13-cis retinoic acid	I	Pediatric: Refractory solid tumors or leukemias	Vorinostat 230 mg/m^2^/dose and vorinostat 180 mg/m^2^/dose 4× per week and 13cRA 80 mg/m^2^/dose 2× daily, days 1–14 every 28 days	Single agent: neutropenia, thrombocytopenia, and hypokalemia	Prolonged stable disease in 1/7 with diffuse intrinsic pontine glioma (DIPG)	(62)
				Combination therapy: thrombocytopenia, neutropenia, anorexia, and hypertriglyceridemia		

Vorinostat and temozolomide	I	Pediatric: Relapsed or refractory primary brain or spinal cord tumors	Vorinostat 300 mg/m^2^/day and temozolomide 150 μg/m^2^/day, 5-day cycles every 28 days	Myelosuppression	Stable disease in 1/7 with high-grade glioma (HGG)	(63)

Valproic acid	I	Pediatric: Refractory solid or CNS tumors	Valproic acid 3× daily to maintain rough concentrations of 75–100 μg/mL	None	Response in 2/4 with DIPG (1 partial and 1 minor)	(66)

Vorinostat and bortezomib	I	Pediatric: Refractory or recurrent solid tumors (6/23 malignant glioma)	Vorinostat 230 mg/m^2^/day, days 1–5 and 8–12 of 21-day cycle, bortezomib 1.3 mg/m^2^/day on days 1, 4, 8, and 11 of a 21-day cycle	Sensory neuropathy, nausea, anorexia	No objective responses observed	(65)

Panobinostat and bevacizumab	I	Adult: Recurrent HGG	Panobinostat 30 mg 3× per week, every other week, with bevacizumab 10 mg/kg every other week	None	3/12 partial response, 7/12 stable disease	(72)

Vorinostat, bevacizumab and irinotecan	I	Adult: Recurrent glioblastoma	Vorinostat 400 mg twice daily on days 1–3 and 15–17, every 28 days	Fatigue, hypertension/hypotension, and central nervous system ischemia	Overall survival 7.3 months	(83)

Vorinostat and isotretinoin, or vorinostat and isotretinoin and carboplatin	I	Adult: Recurrent malignant glioma	Vorinostat 400 mg/day, days 1–14, isotretinoin 100 mg/m^2^/day, days 1–21	Elevated AST, hypertriglycidemia	Progression-free survival at 6 months in 10/55 patients (7/10 had glioblastoma)	(84)
			Carboplatin excessive toxicity, replaced with temozolomide. Vorinostat 500 mg/day, days 1–7 and 15–21, isotretinoin 100 mg/m^2^/day, days 1–21, temozolomide 150 mg/m^2^/day, days 1–7 and 15–21	None		

Panobinostat with fractionated stereotactic re-irradiation therapy	I	Adult: HGG	Panobinostat 30 mg 3× weekly during radiotherapy. Radiation dose was 35 in 3.5 Gy fractions given over 2 weeks	Thrombocytopenia, neutropenia, prolonged QTc	Progression-free survival at 6 months in 30 mg cohort, 5/6 patients. Median overall survival in 30 mg cohort 16.1 months	(74)

Vorinostat and temozolomide	I	Adult: HGG	Vorinostat 500 mg days 1–7 and 15–21 of every 28-day cycle in combination with temozolomide150 mg/m^2^/day days 1–5 of every 28-day cycle	Anorexia, alternative lengthening of telomeres rise, thrombocytopenia, hemorrhage	Not specified	(85)

**Table 2:** **Summary of phase II clinical trials investigating histone deacetylase inhibitors in pediatric and adult high-grade glioma (HGG)**.

Clinical trials	Phase	Population	Drug regimen	Side effects	Results	Reference
Panobinostat	II	Adult: Recurrent HGG	Panobinostat 30 mg 3× per week, every other week, with bevacizumab 10 mg/kg every other week	Bone marrow toxicity and hypophosphatemia	Glioblastoma arm closed at interim analysis, median overall survival 9 months (range 6–19 months). Anaplastic glioma arm to completion, median overall survival 17 months (range 5–27 months)	(73)

Vorinostat	II	Adult: Recurrent glioblastoma, receiving ≤1 chemotherapy regimes for progressive disease	200 mg 2× daily for 14 days, then 7-day rest	Thrombocytopenia, fatigue, hyponatremia, dehydration	Median overall survival 5.7 months (range 0.7–28+ months), 9/52 patients progression free at 6 months with median duration of stable disease 11.2 months (range 6.8–28+ months)	(69)

Vorinostat and bortezomib	II	Adult: Recurrent glioblastoma	400 mg daily for 14 days of a 21-day cycle, 1.3 mg/m^2^ bortezomib days 1, 4, 8, and 11	Bone marrow toxicity, fatigue, neuropathy	0/34 progression free at 6 months	(70)

Romidepsin	I/11	Adult: Recurrent HGG	13.3 mg/m^2^/day on days 1, 8, and 15 of each 28-day cycle	Bone marrow toxicity and fatigue	Median overall survival 34 weeks (95% confidence interval 21–47 weeks)	(71)

Radiotherapy with temozolomide and valproic acid	II	Adult: Newly diagnosed glioblastoma	Valproic acid, 25 mg/kg, 2× daily. First valproic acid dose 1 week before the first day of radiotherapy at 10–15 mg/kg/day	Bone marrow toxicity, neurological toxicity, metabolic toxicity	Median overall survival 29.6 months (range 21–63.8 months)	(75)

Vorinostat, temozolomide, and radiotherapy	I/II	Adult: Newly diagnosed glioblastoma	Vorinostat 300 mg/day, days 1–5 weekly during radiotherapy and with temozolomide, after 4–6 weeks break, up to 12 cycles of vorinostat 400 mg/day, days 1–7 and 15–21 with temozolomide	Neutropenia, thrombocytopenia and lymphopenia	Time to progression 8.05 months (95% confidence interval 6.21–9.30)	(64)

Vorinostat, bevacizumab, and temozolomide	I/II	Adult: Recurrent malignant glioma	Vorinostat 400 mg/day, days 1–7 and 15–21 of each 28-day cycle, temozolomide daily dosing at 50 mg/m^2^/day, bevacizumab 10 mg/kg every other week starting day 1	Bone marrow toxicity, seizure, venous thromboembolism	Median overall survival 12.5 months (95% confidence interval 8.8–14.3 months)	(82)

A second phase I study by the Children’s Oncology Group investigated vorinostat with the alkylating agent temozolomide in relapsed or refractory primary CNS tumors and showed that 300 mg/m^2^/day of vorinostat in combination with 150 mg/m^2^/day of temozolomide is well tolerated in 5-day cycles every 28 days (70). Myelosuppression was the major dose-limiting toxicity (70). Stable disease was observed in one of seven patients with HGG (70). An ongoing phase I/II trial for newly diagnosed glioblastoma is investigating vorinostat with radiotherapy and concomitant temozolomide (71).

A further phase I trial conducted by the Children’s Oncology Group investigated vorinostat in combination with bortezomib, a selective inhibitor of the ubiquitin–proteasome pathway, in children with recurrent or refractory solid tumors (72). This showed a MTD of vorinostat 230 mg/m^2^/day on days 1–5 and 8–12 of a 21-day cycle and bortezomib 1.3 mg/m^2^ on days 1, 4, 8, and 11 of the same cycle (72). Dose-limiting toxicities included sensory neuropathy, nausea, and anorexia (72). Six of twenty-three patients had a malignant glioma; no objective response was observed in any of the patients (72).

The Children’s Oncology Group has also conducted a phase I trial investigating the HDAC inhibitor valproic acid in children with refractory solid or CNS tumors (73). They showed that valproic acid administered three times daily to maintain trough concentrations of 75–100 μg/mL was well tolerated by patients. No dose-limiting toxicities were observed at this dose. Of four patients with DIPG, one patient (glioblastoma) was observed to have a confirmed partial response and one patient (DIPG) was observed to have a minor response (73).

Ongoing clinical trials are investigating event-free survival in children with newly diagnosed HGGs and brainstem gliomas, using valproic acid with radiotherapy, followed by bevacizumab (74). A phase II/III trial is ongoing having recruited children with HGG and is studying the event-free survival using vorinostat, or temozolomide, or bevacizumab in combination with radiotherapy, followed by treatment with bevacizumab and temozolomide (75).

### Adult HGG Trials Using HDAC Inhibitors

The North Central Cancer Treatment Group has conducted two phase II trials looking at vorinostat as a treatment for glioblastoma (76, 77). The first trial using vorinostat as a single agent found that it had modest activity in patients with recurrent glioblastoma (76). A cohort of 66 patients was treated, and median overall survival from study entry was 5.7 months (range 0.7–28+ months) with a median time to progression of 1.9 months (range 0.3–28+ months). Interestingly, 15% of patients were progression free at 6 months, and their duration of disease stability was long with a median of 11.2 months (range 6.8–28+ months) (76). This study revealed a subpopulation who benefit from HDAC inhibitor therapy, with the potential to investigate vorinostat in combination with other therapies, as well as its effect on newly diagnosed glioblastoma (76).

The second trial by the North Central Cancer Treatment Group investigated vorinostat in combination with the proteasome inhibitor bortezomib, in patients with recurrent glioblastoma (77). Unfortunately, in this trial, the progression-free survival at 6 months was 0%, median time to progression was 1.5 months (range 0.5–5.6 months), and median overall survival was 3.2 months (77). Therefore, this combination was not recommended for further investigation in patients with recurrent glioblastoma (77).

A phase I/II trial using the HDAC inhibitor romidepsin was conducted by the North American Brain Tumor Consortium for adults with recurrent malignant glioma (78). Thirty-five patients with recurrent glioblastoma were entered to the study, and the median overall survival was 34 weeks (95% CI, 21–47 weeks) (78). This showed that there was no significant clinical activity of romidepsin as a single agent in unselected patients with recurrent glioblastoma, and so it was concluded to be ineffective (78).

Panobinostat is an HDAC inhibitor with anti-angiogenic activity and has been tested in a phase I trial with the anti-VEGF monoclonal antibody bevacizumab, in patients with recurrent HGG (79). The recommended doses were oral panobinostat 30 mg three times per week, every other week, with bevacizumab 10 mg/kg every other week (79). The major dose-limiting toxicity was thrombocytopenia (79). The trial was escalated to phase II for patients with recurrent glioblastoma and recurrent anaplastic glioma; however, of 24 patients with glioblastoma, median overall survival was 9 months (range 6–19 months), and the glioblastoma cohort of the trial was closed at interim analysis (80). The trial was completed in anaplastic glioma patients, with a median overall survival of 17 months (range 5–27 months) (80). Panobinostat in combination with bevacizumab was concluded to be no more effective than bevacizumab alone; this is hypothesized to be due to diminished transport of panobinostat and bevacizumab across the blood–brain barrier (80). Preclinical evidence has shown that panobinostat may act as a radiosensitizer, and recently, a phase 1 trial combining panobinostat with stereotactic re-irradiation in patients with recurrent HGG has been reported (81). The results were more promising than panobinostat with bevacizumab, with a progression-free survival at 6 months of 83% in the panobinostat and stereotactic re-irradiation therapy group, compared to 30.4% in the panobinostat with bevacizumab group (80, 81). It would be interesting to investigate this further *via* a phase II trial and assess the efficacy of the synergistic relationship between panobinostat and fractionated stereotactic re-irradiation therapy.

Valproic acid is an antiepileptic drug with HDAC inhibitor activity. A phase II trial investigated concurrent radiotherapy, temozolomide, and valproic acid in 37 patients with newly diagnosed glioblastoma (82). Median overall survival was 29.6 months (range 21–63.8 months) (82). Compared to five other phase II trials investigating radiotherapy and temozolomide with radiation modifiers including erlotinib, enzastaurin, and poly-ICLC, this result shows a large increase in overall survival, with the other studies showing overall survivals from 8.6 to 9.3 months (83–87). This observation is further supported by a retrospective study conducted by Weller et al. Patients with glioblastoma receiving radiotherapy alone or radiotherapy with temozolomide were analyzed to investigate the effect of antiepileptic therapy given during this treatment time (88). They found the overall survival was similar for patients taking an antiepileptic drug versus those who were not, except for those taking the HDAC inhibitor valproic acid (88). Patients taking valproic acid had a better overall survival benefit from radiotherapy with temozolomide (hazard ratio 0.39, 95% confidence interval 0.24–0.63), compared to those taking another antiepileptic drug or no antiepileptic drug (88).

A phase I/II trial investigated the efficacy of vorinostat in combination with bevacizumab and daily temozolomide in recurrent glioblastoma (89). Dose-limiting toxicities included bone marrow toxicity, hyperglycemia, pulmonary embolism, bowel perforation, and intracranial hematoma (89). The 6-month progression-free survival was 52.4% (95% CI, 36.4–66.1%) (89). The best radiographic responses were 2 complete responses, 17 partial responses, and 20 stable responses, with 1 radiographic progression (89). This trial provides promising insight into the efficacy of bevacizumab, temozolomide, and vorinostat on recurrent glioblastoma with reasonable toxicity (89).

Histone deacetylase inhibitors as a monotherapy for HGGs seem disappointing; however, the latest trials investigating HDAC inhibitors as part of a combination therapy seem promising in improving prognosis in this difficult to treat malignancy, with further ongoing studies that are yet to be published (81, 90–92).

## Future Directions

### Histone Demethylase Inhibition in Experimental Models

With increasing knowledge of oncogenic epigenetic changes underlying HGGs, such as the H3.3 K27M mutation, targeting to reverse these changes has been explored, although to date the field is far less advanced than HDAC inhibition. Hashizume et al. explored inhibiting JMJD3, the H3K27 demethylase, with GSKJ4 as a therapeutic strategy for pediatric HGG (93). By increasing H3K27 methylation, this could inhibit gene expression that would drive gliomagenesis, as well as blocking cell differentiation (94). H3.3 K27M glioma cell lines showed 50% growth reduction, more apoptosis, and complete inhibition of clonal growth with GSKJ4 treatment, while JMJD3 depleted glioma cell lines showed no significant reduction in proliferation (93, 94). In athymic (nu/nu genotype, BALB/c background) mice with brainstem K27M glioma xenografts, GSKJ4 treatment resulted in significant tumor growth reduction and extended survival (93, 94).

### Combination Therapy Is Synergistic

With the knowledge of H3.3 mutations and subsequent aberrant histone methylation, a recent study by Grasso et al. has investigated the use of the pan-HDAC inhibitor panobinostat, with the histone demethylase inhibitor GSKJ4 (95). They used a panel of 14 patient-derived DIPG cell cultures, obtained from both biopsy and autopsy samples (95). Western blot analyses of cells expressing the H3.3 K27M mutation showed that following panobinostat treatment, there was an increase in H3 acetylation and H3K27 methylation, suggesting there is a partial rescue of the H3.3 K27M-induced global hypomethylator phenotype CHOP (95). Furthermore, they showed that panobinostat was synergistic with GSKJ4 in decreasing cell viability of the H3.3 K27M mutant DIPG cells (95). This presents an exciting option to target histone methylation and acetylation in clinical trials with the hope of combating HGGs.

As well as targeting HDAC inhibition, EZH2 inhibition provides an alternative mechanism to prevent aberrant histone methylation of target genes, which may promote cell differentiation and prevent cell proliferation in several tissues (96). This has been demonstrated by preclinical studies in pediatric rhabdomyosarcoma (97). By using pharmacological inhibition of EZH2, the aggressiveness of rhabdomyosarcoma is less with a more differentiated phenotype (97). This provides further treatment options for rhabdomyosarcoma by using EZH2 inhibitors as adjuvant therapy, thus with a likely possibility of increasing the effectiveness of current conventional treatment (97). EZH2 overexpression is reported in many malignancies including lymphoma, breast cancer, and prostate cancer (98–100). Phase I pediatric and phase II adult clinical trials are underway to investigate the efficacy of EZH2 inhibitors in hematological malignancies, as well as genetically defined solid tumors, including mesothelioma and malignant rhabdoid tumors (ClinicalTrials.gov identifier: NCT 02601937 and NCT 02601950).

Although this review has focused predominantly on aberrations in histone H3K27 methylation, aberrations in methylation and acetylation of other histone proteins may provide positive or negative prognostic indicators for patients with glioma. Liu et al. reported the relationship between multiple histone modifications and patient prognosis (101). They analyzed by recursive partitioning analysis, a retrospective cohort of patients with HGG, with progression-free survival and overall survival as the primary end points (101). Immunohistochemical analysis of H3K4, H4R3, H4K20, H3K9, H3K18, H4K12, and H4K16 from 230 surgical HGG specimens suggested that lower levels of histone H3K4 methylation were associated with poor prognosis (101). In contrast to this, they found lower levels of histone acetylation in H3K18 were associated with a more favorable survival (101). This study highlights the potential prognostic impact of epigenetic changes in patients with HGG. This may provide future direction in selecting patients for optimal adjuvant treatment.

Finally, the targeting of genetic mutations in combination with epigenetic aberrations may increase the likelihood of successfully treating adult and pediatric HGG. Taylor et al. identified 21% of pediatric DIPG harbored heterozygous somatic coding mutations in the gene *ACVR1*, which encodes the activin A type I receptor serine/threonine kinase ALK2 (102). *ACVR1* mutations were found to cosegregate with histone H3.1 K27M mutated DIPG (102). Previously, in patients with the autosomal dominant congenital childhood developmental disorder fibrodysplasia ossificans progressiva, identical *ACVR1* mutations have been shown to constitutively activate the bone morphogenic protein (BMP)-dependent transforming growth factor-β pathway (103). The results of this study suggest a role for BMP inhibitors to target one of the possible mechanisms driving tumorigenesis in DIPG (102). Future trials would be of interest to see the efficacy of BMP inhibitors singly or in combination with epigenetic targeting therapies such as HDAC or EZH2 inhibitors.

### Limitations of Therapeutic Targeting of Epigenetics in HGG

The discovery of aberrant histone modifications propagating gliomagenesis has allowed the exploration of HDAC inhibitors and histone demethylase inhibitors in an attempt to combat an aggressive brain tumor. Current limitations of epigenetic targeting remain a challenge; in particular, the mechanism of HDAC inhibitors and their effect on cellular signaling pathways remains to be elucidated, and the effects of broadly altering functional epigenetic changes is unpredictable. Furthermore, there is intratumoral genetic heterogeneity, which may protect HGGs from being fully eradicated, as well as altering the uptake and concentration of the HDAC inhibitor into the cells. Furthermore, methods are yet to be found, which allow better penetration of HDAC inhibitors through the blood–brain barrier. For example, the HDAC inhibitors panobinostat and vorinostat are substrates for the major efflux transporters at the blood–brain barrier, which may give some rationale as to why they have so far failed to translate into effective therapies in clinical trials (104). The above reasons are likely to contribute to the slow progress of clinical trials investigating HDAC inhibitor use in HGGs.

Targeting multiple epigenetic and genetic aberrations will likely be the key to succeeding in treating HGGs, and future clinical trials are needed to further explore combination therapies, alongside novel techniques to improve the penetration of the blood–brain barrier.

## Author Contributions

MW, WS, and KK: manuscript research and writing. SL and KM: manuscript review and revision.

## Conflict of Interest Statement

The authors declare that the research was conducted in the absence of any commercial or financial relationships that could be construed as a potential conflict of interest.
